# Molecular and phylogenetic characterization of the sieve element occlusion gene family in *Fabaceae *and non-*Fabaceae *plants

**DOI:** 10.1186/1471-2229-10-219

**Published:** 2010-10-08

**Authors:** Boris Rüping, Antonia M Ernst, Stephan B Jekat, Steffen Nordzieke, Anna R Reineke, Boje Müller, Erich Bornberg-Bauer, Dirk Prüfer, Gundula A Noll

**Affiliations:** 1 Institut für Biochemie und Biotechnologie der Pflanzen, Westfälische Wilhelms-Universität Münster, Hindenburgplatz 55, D-48143 Münster, Germany; 2 Fraunhofer Institute for Molecular Biology and Applied Ecology (IME), Forckenbeckstraße 6, D-52074 Aachen, Germany; 3 Institut für Evolution und Biodiversität, Westfälische Wilhelms-Universität Münster, Hüfferstraße 1, D-48149 Münster, Germany

## Abstract

**Background:**

The phloem of dicotyledonous plants contains specialized P-proteins (phloem proteins) that accumulate during sieve element differentiation and remain parietally associated with the cisternae of the endoplasmic reticulum in mature sieve elements. Wounding causes P-protein filaments to accumulate at the sieve plates and block the translocation of photosynthate. Specialized, spindle-shaped P-proteins known as forisomes that undergo reversible calcium-dependent conformational changes have evolved exclusively in the *Fabaceae*. Recently, the molecular characterization of three genes encoding forisome components in the model legume *Medicago truncatula *(*MtSEO1*, *MtSEO2 *and *MtSEO3*; SEO = sieve element occlusion) was reported, but little is known about the molecular characteristics of P-proteins in non-*Fabaceae*.

**Results:**

We performed a comprehensive genome-wide comparative analysis by screening the *M. truncatula*, *Glycine max*, *Arabidopsis thaliana*, *Vitis vinifera *and *Solanum phureja *genomes, and a *Malus domestica *EST library for homologs of *MtSEO1*, *MtSEO2 *and *MtSEO3 *and identified numerous novel *SEO *genes in *Fabaceae *and even non-*Fabaceae *plants, which do not possess forisomes. Even in *Fabaceae *some *SEO *genes appear to not encode forisome components. All *SEO *genes have a similar exon-intron structure and are expressed predominantly in the phloem. Phylogenetic analysis revealed the presence of several subgroups with *Fabaceae*-specific subgroups containing all of the known as well as newly identified forisome component proteins. We constructed Hidden Markov Models that identified three conserved protein domains, which characterize SEO proteins when present in combination. In addition, one common and three subgroup specific protein motifs were found in the amino acid sequences of SEO proteins. *SEO *genes are organized in genomic clusters and the conserved synteny allowed us to identify several *M. truncatula *vs *G. max *orthologs as well as paralogs within the *G. max *genome.

**Conclusions:**

The unexpected occurrence of forisome-like genes in non-*Fabaceae *plants may indicate that these proteins encode species-specific P-proteins, which is backed up by the phloem-specific expression profiles. The conservation of gene structure, the presence of specific motifs and domains and the genomic synteny argue for a common phylogenetic origin of forisomes and other P-proteins.

## Background

In vascular plants, photoassimilates are transported through differentiated sieve elements (SEs) in the phloem forming a network of sieve tubes throughout the plant [1]. The pressure-driven mass flow [2] requires a high degree of functional specialization of the phloem during development. In order to enable efficient translocation of photoassimilates, SEs loose most of their organelles and thus the ability to perform protein biosynthesis [3]. Mature SEs are therefore dependent on adjacent, metabolically-active companion cells, which are connected to SEs by so-called pore-plasmodesm units [4]. The pressure within the sieve tubes can reach 30 bar [5], so rapid and efficient protection against wounding is essential and favoured the evolution of a plugging mechanism based on specialized phloem proteins (P-proteins) [6]. These structural proteins accumulate in the cytoplasm of metabolically-active, undifferentiated SEs, but are anchored to the plasma membrane when SEs mature [7]. After wounding, they detach from their parietal location and plug downstream sieve plates by forming a gel-like mass, thereby preventing the loss of photoassimilates [8]. This occurs in all the dicotyledonous plant families that have been studied, and P-proteins have also been identified in certain monocotyledonous plants [9]. There is currently no standardized classification for P-proteins although different types were often distinguished by their tubular, fibrillar, granular or crystalline ultrastructure, which may represent different developmental or conformational states of the same protein subunits rather than evolutionarily-distinct families [10]. Nevertheless, *Fabaceae *plants possess a special type of elongated crystalline P-protein bodies [11], which show a unique type of reactivity. The spindle shaped protein bodies, also known as forisomes ("gate bodies") [12], are able to undergo a reversible, calcium-induced conformational change and can consequently plug and open the sieve elements after wounding and regeneration. Three SEO (sieve element occlusion) proteins named MtSEO1, MtSEO2 and MtSEO3 have been identified in the model legume *Medicago truncatula*, and their role in forisome structure and assembly has been confirmed by immunological and GFP-fusion studies [13-15]. Comprehensive promoter analyses in *M. truncatula *roots and *Nicotiana tabacum *plants demonstrated a restricted expression of the corresponding *MtSEO *genes in immature sieve elements [14,16], indicating a highly conserved regulation of promoter activities among diverse plant species, including non-*Fabaceae *lacking forisomes.

Although genes encoding forisome components of *Fabaceae *have recently been isolated and characterized, little is known about the genetic basis of structural P-proteins in other plant families. The only P-protein to be characterized thus far is phloem protein 1 (PP1) from *Cucurbita maxima *[17]. Immunological studies identified this filamentous protein in SE slime plugs and P-protein bodies, although the corresponding mRNA was shown to accumulate in companion cells [18].

In this study, we report the identification of several new *SEO *genes in *Fabaceae *and, most interestingly, also in non-*Fabaceae *plants (which do not possess forisomes). The unexpected occurrence of *SEO *genes in plant families lacking forisomes may argue that these genes encode other structural P-proteins, which implies a common phylogenetic origin. To further characterize the newly-identified *SEO *genes, we analyzed their gene structure and genomic synteny using bioinformatics and studied their expression by RT-PCR. Selected *SEO *genes were also studied by promoter analysis in transgenic plants.

## Results

### The *SEO *gene family in *Fabaceae*

**Figure 1:**
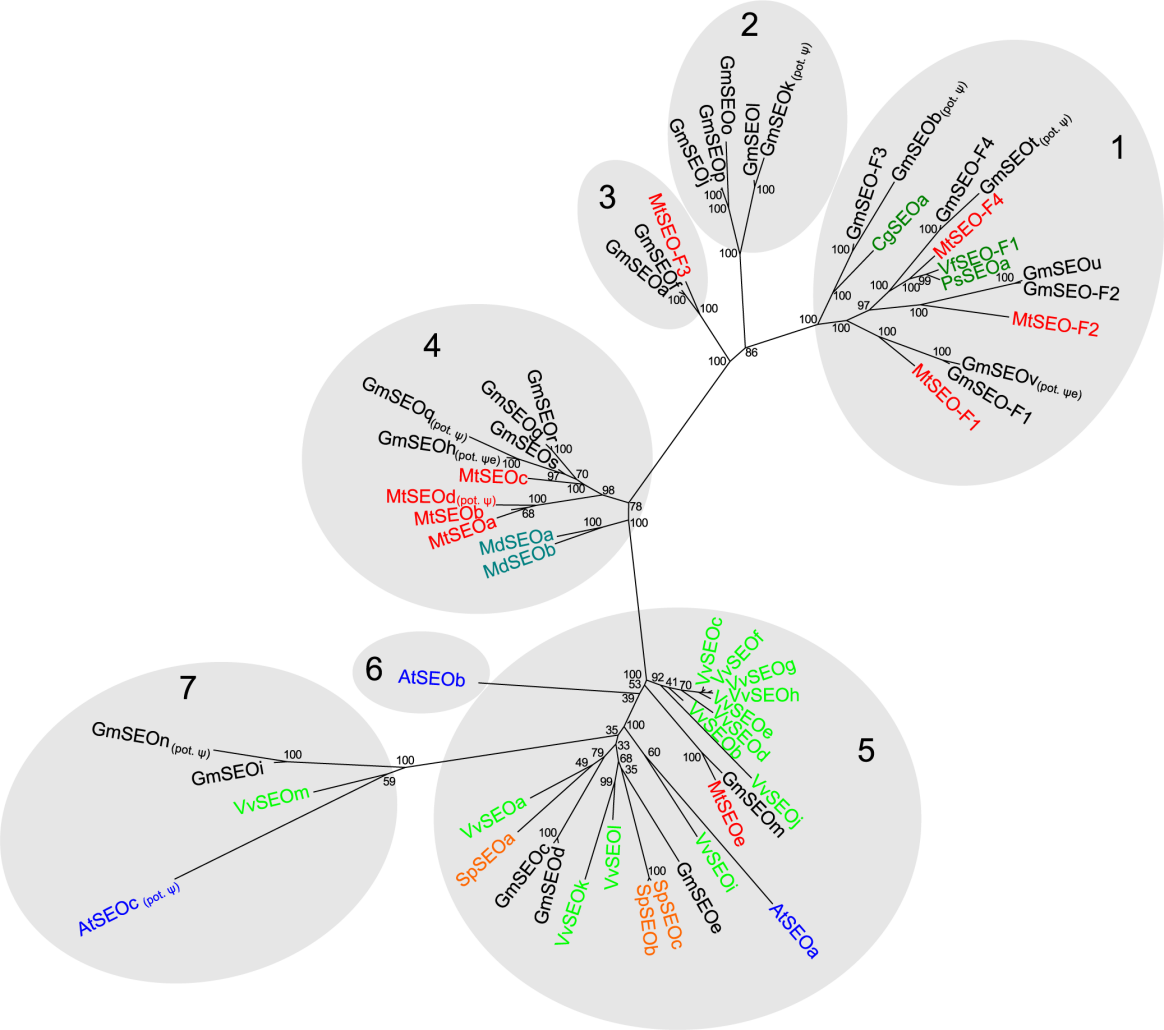
**Maximum likelihood phylogenetic tree of SEO proteins from different plants**. The phylogenetic tree was constructed with RAxML from a T-Coffee protein sequence alignment and with a bootstrap support of 1000 replicates. Bootstrap percentages are shown on the nodes. Branch lengths are proportional to the number of amino acid substitutions. The shaded parts of the tree represent the subgroups identified with OrthoMCL. Mt = *Medicago truncatula*, Gm = *Glycine max*, Vf = *Vicia faba*, Cg = *Canavalia gladiata*, Ps = *Pisum sativum*, Md = *Malus domestica*, At = *Arabidopsis thaliana*, Vv = *Vitis vinifera*, Sp = *Solanum phureja*, pot. ψ = potential pseudogene, pot. ψe = expressed potential pseudogene.

BLAST searches were carried out using the nucleotide sequences (and derived amino acid sequences) of the three known *M. truncatula SEO *genes [14,15], identifying six further candidate *SEO *genes in the *M. truncatula *genome and 26 in *Glycine max*. In order to determine whether or not these genes are expressed, we amplified a 1-kbp cDNA fragment from each gene by RT-PCR using total seedling RNA as the template. This generated products for five of the six newly-identified *M. truncatula *genes and 21 of the 26 *G. max *genes, the remaining six genes being identified as potential pseudogenes ('pot. ψ' in Figure 1; Additional file 1). Full-length cDNAs were produced for the expressed genes by PCR using gene-specific primers. The potential pseudogenes were tested by RT-PCR using a collection of different primer combinations and total RNA from young and old leaves, shoots, roots, buds and flowers, with no expression detected (data not shown). Two of the expressed genes from *G. max *were reclassified as potential expressed pseudogenes ('pot. ψe' in Figure 1) because sequencing revealed the presence of frameshift mutations in their open reading frames, causing premature termination of protein synthesis. In order to include all pseudogenes in subsequent phylogenetic analyses, full-length cDNA sequences were generated *in silico *using the procedures described in the Methods section.

Next, we set out to establish whether any of the newly-identified SEO proteins were present in the forisomes of either *M. truncatula *or *G. max*. Purified forisomes from each species were separated by SDS-PAGE and peptide sequences were generated for the 75-kDa protein band by ESI-MS/MS following established protocols [12-14]. Peptide sequences derived from the *M. truncatula *forisomes (Additional file 2) indicated the presence of one further SEO protein in addition to the known components MtSEO1-3 [13-15]. Similarly, four of the 26 *G. max *SEO proteins were identified in *G. max *forisomes, where no components had been previously identified (Additional file 2). These results suggest that many of the *SEO *genes do not encode forisome component proteins, although it is possible that they are present but at levels below our detection threshold. In order to distinguish SEO proteins known to encode forisome components from other SEO proteins, we propose that MtSEO1-3 should be renamed MtSEO-F1, MtSEO-F2 and MtSEO-F3, and that the newly identified component should be named MtSEO-F4 (**SEO-F**, **S**ieve **E**lement **O**cclusion by **F**orisomes). The four SEO proteins found in purified *G. max *forisomes should similarly be designated GmSEO-F1-F4. For all other *Fabaceae *SEO proteins (and SEO proteins from other plant families, see below) whose function is currently unknown, we recommend the temporary assignment of a lower case letter (SEOa, SEOb, etc) until their presence in the forisome can be confirmed (in which case they will be assigned an SEO-F number) or another function is determined (in which case additional functional categories can be introduced).

### The *SEO *gene family in non-*Fabaceae*

Using the *Fabaceae SEO *sequences described above, we screened the genomes of several dicotyledonous non-*Fabaceae *plants, i.e. *Arabidopsis thaliana *(*Brassicaceae*), *Vitis vinifera *(*Vitaceae*) and *Solanum phureja *(*Solanaceae*) as well as an EST collection for *Malus domestica *(*Rosaceae*). This identified three *A. thaliana *genes (designated *AtSEOa-c*), 13 *V. vinifera *genes (designated *VvSEOa-m*) and three *S. phureja *genes (designated *SpSEOa-c*) (Additional file 1). Two full-length *SEO *genes, designated *MdSEOa *and *b *(Additional file 1), and further partial fragments were found in the *M. domestica *EST collection. Potential *SEO *gene fragments of several other angiosperm plants could also be identified by BLAST search in NCBI GenBank (data not shown). In contrast, no *SEO *genes were identified in the yet sequenced genomes of the monocotyledons *Oryza sativa, Brachypodium distachyon*, *Zea mays *and *Sorghum bicolor *nor in the moss *Physcomitrella patens*. RT-PCR confirmed that all the *A. thaliana*, *S. phureja *and *M. domestica *genes were expressed with the exception of *AtSEOc*, which appears to be a pseudogene. The expression profiles of *VvSEOa-m *were not determined due to the lack of the sequenced genotype in our laboratories.

### Phylogenetic relationships among *SEO *genes from *Fabaceae *and non-*Fabaceae *plants

The phylogenetic relationships among the *SEO *genes were calculated by creating a maximum likelihood tree from an alignment of all SEO protein sequences. To provide further support for the tree topology we clustered the proteins into subgroups with OrthoMCL (Figure 1). The recently reported forisome protein VfSEO-F1 (formerly known as VfFOR1) from *Vicia faba *[14] and the potential forisome proteins CgSEOa (formerly known as CgFOR1) from *Canavalia gladiata *[14] and PsSEOa (formerly known as PsSEO1) from *Pisum sativum *[19] were also included in the phylogenetic tree. With the exception of MtSEO-F3, all SEO-F proteins clustered in subgroup 1. In addition, several potential pseudogenes as well as one GmSEO protein (GmSEOu) clustered within this group. It should be noted that GmSEOu and GmSEO-F2 share several identical forisome-specific peptide sequences, so GmSEOu is likely to be involved in the formation of forisomes. Subgroups 2 and 3, found on the same branch of the tree, contain predominantly *G. max *SEO proteins of yet unknown function (the only exception is MtSEO-F3). Thus, subgroups 1-3 appear to be *Fabaceae*-specific. Subgroup 4 contains nine *Fabaceae *SEO proteins and both SEO proteins (MdSEOa and b) from the closely related *Rosaceae*, whereas subgroup 5 contains SEO proteins from all plant families included in the study that have sequenced genomes. AtSEOa is the only member of subgroup 6, which is closely related to subgroup 5. Subgroup 7 contains SEO proteins from *G. max, V. vinifera *and *A. thaliana*.

**Figure 2:**
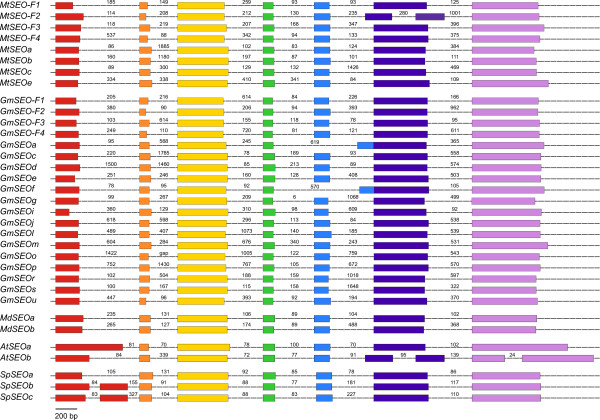
**Schematic overview of *SEO *gene exon-intron structure**. Exons are represented as colored boxes, introns as dashed lines. Introns are not drawn to scale, but the length of the individual introns is indicated in base pairs. Mt = *Medicago truncatula*, Gm = *Glycine max*, Md = *Malus domestica*, At = *Arabidopsis thaliana*, Sp = *Solanum phureja*.

In addition to their high degree of amino acid similarity (>30%), most *SEO *genes have a conserved exon-intron structure as shown in Figure 2, the exceptions being *GmSEOa *and *GmSEOf *(one intron missing) and *MtSEO-F2*, *AtSEOb*, *SpSEOb *and *SpSEOc *(additional introns).

### Expression profiles and promoter activities of the *SEO *genes

**Figure 3:**
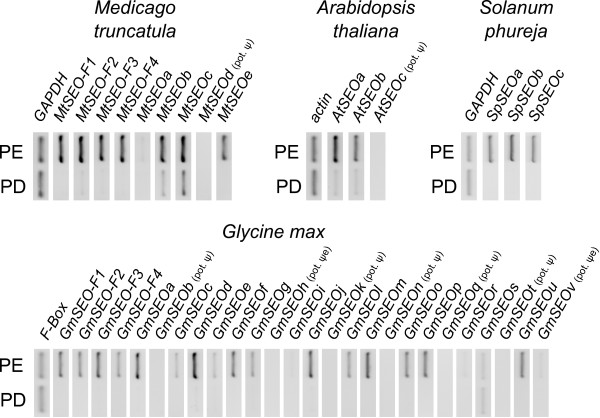
**RT-PCR analysis of *SEO *gene expression in *M. truncatula*, *G. max*, *A. thaliana *and *S. phureja***. *SEO *genes were amplified from cDNA prepared from total RNA isolated from phloem-enriched (PE) and phloem-deficient (PD) tissue. The constitutively expressed *ACT2 *(for *A. thaliana*), *GAPDH *(for *M. truncatula*, *S. phureja*) and *F-box *(for *G. max*) genes served as positive controls. The integrity of all PCR products was verified by sequencing.

The expression of *MtSEO-F1-3 *was recently shown to be restricted to immature sieve elements [14,16]. To gain an initial impression of whether the *MtSEO*, *GmSEO*, *AtSEO *and *SpSEO *genes were also expressed in the phloem we performed RT-PCRs using total RNA from phloem-enriched and phloem-deficient tissue (see Methods). With the exception of gene *GmSEOs*, mRNA levels for all the *SEO *genes were significantly higher in the phloem-enriched tissues (Figure 3). As expected, no mRNA was detected for the potential pseudogenes *MtSEOd*, *AtSEOc*, *GmSEOb*, *GmSEOk*, *GmSEOn*, *GmSEOq *and *GmSEOt*. Transcripts for the potential expressed pseudogenes *GmSEOh *and *GmSEOv *harboring frameshift mutations were also detected, although it should be noted that *GmSEOh *mRNA was only found in roots (data not shown). The constitutively expressed *ACT2 *(for *A. thaliana*), *GAPDH *(for *M. truncatula *and *S. phureja*) and *F-box *(for *G. max*) genes served as positive controls, as they have been found to be the most appropriate control genes for the plant species included in this study (see Methods).

**Figure 4:**
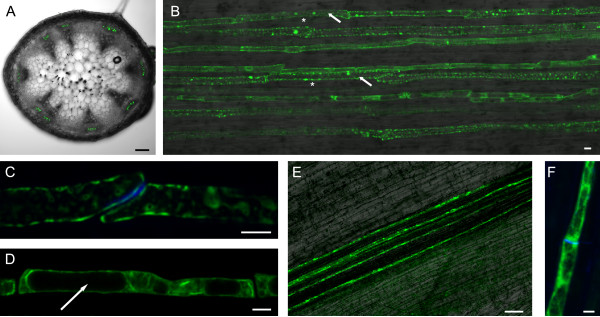
**Analysis of the *AtSEOa *and *GmSEO-F1 *promoter in transgenic plant tissue**. CLSM detection of GFP_ER _activity in P*AtSEOa*-GFP_ER _transgenic *A. thaliana *plants (A to D) and P*GmSEO-F1*-GFP_ER _transgenic *G. max *roots (E, F). (A) Transverse sections through an *A. thaliana *stem showing GFP_ER _restricted to the phloem. (B) Overlay of fluorescent and transmitted light images of a longitudinal *A. thaliana *stem section showing GFP_ER _fluorescence in sieve elements (arrows). Non-fluorescent companion cells are marked with an asterisk. (C) Sieve plate of two end-to-end connected fluorescent sieve elements stained with aniline-blue. (D) Sieve element containing large vacuoles, indicated by the white arrow. (E) Longitudinal section through the vascular cylinder of a transgenic *G. max *root. (F) Sieve element with aniline-blue stained sieve plate. Scale bar = 100 μm in A and E, and 5 μm in B-D and F.

The preliminary analysis above provided evidence for phloem-specific expression, but for conclusive proof we set out to analyze the activity of two *SEO *promoters in transgenic plants. We chose promoters from the forisome gene *GmSEO-F1 *and the none forisome gene *AtSEOa*. A *green fluorescent protein *(*GFP*) gene tagged for retention of the product in the endoplasmic reticulum (ER) was placed under the control of each promoter, producing constructs P*AtSEOa*-GFP_ER _and P*GmSEO-F1*-GFP_ER_. Ten independent transgenic *A. thaliana *plants expressing P*AtSEOa*-GFP_ER _were regenerated and analyzed by confocal laser scanning microscopy (CLSM). In stem sections, GFP_ER _fluorescence was detected in the phloem of the vascular bundle (Figure 4A, B), which appears to be restricted to a 'pipeline-like' assembly of cells within the phloem. These cells display the typical end-to-end connection of sieve elements and the presence of sieve plates was verified by aniline-blue staining (Figure 4C). Within the cytoplasm, one or more vacuoles were clearly visible indicating an immature nature of these cells (Figure 4D). Detailed CLSM analysis of five independent transgenic *G. max *roots expressing P*GmSEO-F1*-GFP_ER _also indicated spatially-restricted promoter activity in sieve elements of the vascular cylinder (Figure 4E) showing the same morphology and characteristics (Figure 4F) as described for P*AtSEOa*-GFP_ER _(Figure 4C, D).

### Domain analysis of SEO proteins

**Figure 5:**
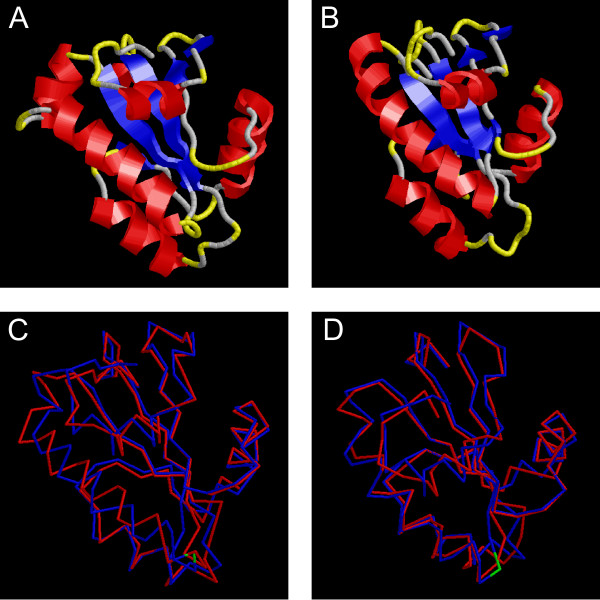
**Predicted thioredoxin fold of MtSEO-F1 and AtSEOa**. Three-dimensional structure of the potential thioredoxin fold in (A) MtSEO-F1 and (B) AtSEOa predicted with I-TASSER. α-helices are coloured in red, β-sheets in blue and turns in yellow. Comparison of the predicted three-dimensional structures of (C) MtSEO-F1 and (D) AtSEOa (both coloured in red) with tryparedoxin II, aligned with TM-align. The structure of tryparedoxin II is coloured in blue, cysteine residues are highlighted in green.

The SEO protein sequences were analyzed to identify any conserved protein domains and gain insights into their relationship with known protein functions, such as the ability of forisomes to respond to calcium. The deduced amino acid sequences of all SEO proteins were screened against the PfamA and Conserved Domain databases [20,21]. No significant hits were found in the PfamA database, but the Conserved Domain database identified "thioredoxin-like" domains in MtSEO-F2, MtSEOa, GmSEOi, GmSEOl and MdSEOa, which cover several subgroups of the SEO family. Based on this result we constructed a specific Hidden Markov Model by aligning all the identified thioredoxin-like domains, and using this to screen the remaining SEO proteins for similar domains. We obtained significant hits for all the SEO proteins and designated the resulting domain as 'potential thioredoxin fold'. Further analysis of the *M. truncatula *and *A. thaliana *'potential thioredoxin fold' domains with the protein structure prediction server I-TASSER, and alignment with known structures in the Protein Data Bank (PDB) with TM-align, revealed that SEO proteins are structurally related to tryparedoxin II (Figure 5), a thioredoxin-like protein [22]. Complete results from I-TASSER and TM-align are provided in Additional file 3. Scanning against predicted proteins from all the plants included in our analysis, the 'potential thioredoxin fold' was also present in several further proteins, including some from monocotyledonous plants, but was not found in *P. patens*. All of these non-SEO proteins contain a PfamA domain belonging to the Pfam-Clan "Thioredoxin-like" indicating that our Hidden Markov Model indeed predicts thioredoxin folds.

**Figure 6:**
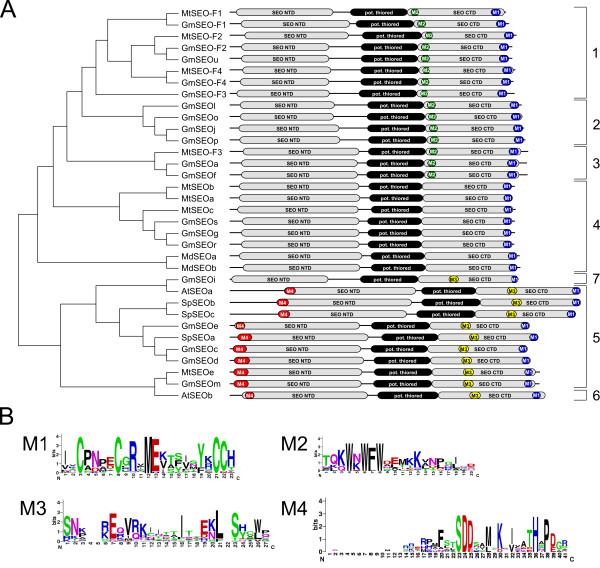
**Domain arrangement of the expressed SEO proteins**. (A) The phylogenetic tree was calculated with RaxML from a T-Coffee protein alignment. Identified protein domains are drawn to scale. The positions of the SEO-NTD and SEO-CTD domains and the potential thioredoxin fold are indicated. (B) Sequence logos: M1 = sequence logo specific for all SEO proteins; M2 = sequence logo specific for SEO subgroups 1-3; M3 = sequence logo specific for SEO subgroups 5, 6 and 7; and M4 = sequence logo specific for SEO subgroups 5 and 6, with potential intrinsic disorder. The position of the individual sequence logos is also indicated in (A).

We also searched for predicted domains using the PfamB database [20], revealing three domains in all SEO proteins. One domain (PB104124) was non-specific and overlapped the two other predicted PfamB domains, and was therefore rejected from further analysis. The second PfamB domain (PB013523) was predicted in the N-terminal part of the proteins and was therefore named SEO-NTD (SEO N-Terminal Domain), whereas the third (PB006891) spanned the C-terminus. Because PB006891 partially overlaps with the 'potential thioredoxin fold' we adjusted the domain by building a new Hidden Markov Model that did not interfere with the fold and subsequently renamed it SEO-CTD (SEO C-Terminal Domain). The domain arrangement of the SEO proteins is shown in Figure 6A. It should be noted that the combination of the two PfamB domains together with the 'potential thioredoxin fold' in a single protein could not be identified in any predicted non-SEO proteins from the analyzed plants and appears unique to the SEO family. However, none of these domains alone is specific for SEO proteins - all three domains are present individually in other proteins, although the PfamB domains were identified in only a few proteins from *M. truncatula *and *G. max *(Additional file 4) and in no proteins from *A. thaliana*, *V. vinifera*, the monocotyledonous plants we analyzed or in *P. patens*. *S. phureja *and *M. domestica *were excluded from this search because gene models were not available.

### Motif search in SEO proteins

Next, we set out to identify motifs (shorter in length than the domains described above) that are specific for the SEO protein family. Therefore, highly-conserved regions were chosen from an alignment of SEO proteins and were used to construct Hidden Markov Model profiles which characterize the motifs. We identified numerous motifs that appeared to be unique to SEO proteins (Figure 6B) as they could not be detected in any other proteins from any of the other plants included in the analysis. The C-terminal M1 motif, containing several conserved cysteine residues, was representative of the entire SEO family, whereas M2 and M3 appeared to be specific for SEO subgroups, perhaps indicating structural and/or functional specialization. The M4 motif, which was 20-35 amino acids in length and matched an in-house database of disordered regions, was found in the N-terminal portion of SEO proteins in subgroups 5 and 6 (Figure 6B) and could reflect intrinsic disorder within this part of the protein. The individual position of the motifs is indicated in Figure 6A.

### Genomic synteny

Finally, we investigated the organization of *SEO *genes in the *M. truncatula*, *G. max*, *A. thaliana *and *V. vinifera *genomes (*M. domestica *and *S. phureja *were excluded due to the lack of annotated genomic data). As shown in Additional file 5, most *SEO *genes appear to be organized in clusters. Seven of nine *M. truncatula SEO *genes (*MtSEO-F1, 2, 4 *and *MtSEOa-d*) are clustered in a 150-kbp segment of chromosome 1, in *G. max*, five *SEO *genes are found in a 50-kbp cluster on chromosome 10, while another six are found in a 55-kbp cluster on chromosome 20. Eight grapevine *SEO *genes are clustered in a 170-kbp segment of chromosome 14 and two of the three *A. thaliana SEO *genes are adjacent on chromosome 3.

**Figure 7:**
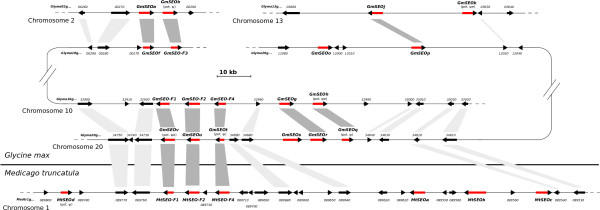
**Genomic synteny of several *SEO *genes from *M. truncatula *and *G. max***. Schematic overview of the genomic synteny of *SEO *clusters on *M. truncatula *chromosome 1 and *G. max *chromosomes 2, 10, 13 and 20. *SEO *genes are shown as red arrows. Other gene models are shown as black arrows. Orthologs between the two *Fabaceae *and paralogs within *G. max *are connected by dark grey shading. Orthologs and paralogs for non-*SEO *genes are indicated by light grey shading.

The organization of the *G. max *genes is worthy of special attention. *GmSEO-F1, 2 *and *4 *- the orthologs of the *M. truncatula *forisome-encoding genes *MtSEO-F1, 2 *and *4 *- are clustered together on a 20-kbp segment of chromosome 10 (Figure 7), but chromosome 20 contains a similarly-arranged cluster comprising the expressed and non-expressed paralogs *GmSEOv*_(potψe)_, *GmSEOu *and *GmSEOt*_(potψ)_. This similar genomic arrangement could also be verified by analyzing the neighboring non-*SEO *related genes. Out of the 26 *GmSEO *genes 11 paralog pairs could be identified (Figure 7 and Additional file 5). Although we isolated *MtSEO-F3 *from cDNA and genomic DNA, we could not identify the sequence in the published *M. truncatula *genome, suggesting a gap in genome coverage. For five out of nine *MtSEO *genes we could identify orthologs in *G. max *(Figure 7 and Additional file 5).

## Discussion

Forisomes are specialized P-proteins found solely in the *Fabaceae*. Genes encoding forisome components were recently identified in *M. truncatula*, and are the founder members of the sieve element occlusion (*SEO*) gene family [13-16]. In this study, we identified many additional *SEO *genes in the genomes of two *Fabaceae *and, interestingly, also in several non-*Fabaceae *plant families (which do not possess forisomes). Even within the *Fabaceae*, it appears that only some of the newly-identified *SEO *genes encode forisome components. Phylogenetic analysis showed that all the known and novel *SEO *genes can be clustered in seven subgroups (Figure 1) and they have a similar exon-intron structure (Figure 2). Their expression is likely to be phloem-specific (Figure 3) most probably restricted to immature sieve elements (SEs) as shown for one *Fabaceae *and one non-*Fabaceae SEO *gene (Figure 4) and the proteins contain highly conserved domains and motifs (Figures 5 and 6).

Forisomes have been described as a special type of P-protein mainly based on their morphological characteristics. Like forisomes, P-proteins of various species were reported to accumulate in immature sieve elements [23] and in their differentiated state both types of P-protein share the same function of blocking sieve elements after phloem injury [8]. Furthermore, forisomes and P-proteins show very similar ultrastructural characteristics in both the condensed and dispersed states [24,25]. A calcium-induced reactivity, as known for forisomes, is also discussed for P-proteins [26,27]. Although the dispersion process is similar in each case, the complete reversibility of the conformational switch is unique to forisomes [12]. It therefore seems likely that the non-forisome *SEO *genes in the *Fabaceae*, and all the *SEO *genes in non-*Fabaceae *plant families, encode other (non-forisome) P-proteins. It should be noted that the *Cucurbita maxima *PP1 protein, which is the only non-forisome P-protein to be characterized thus far, shares neither significant sequence similarities nor any conserved domains with the SEO proteins described herein. For this reason, *PP1 *should not be assigned to the *SEO *gene family, despite its potential functional similarity. Additionally, we and others [18] have not identified any PP1 orthologs in the genomes of non-*Cucurbitaceae *plants, suggesting PP1 may play a unique role in the phloem of the *Cucurbitaceae *family.

We used a number of bioinformatics approaches to identify functional motifs and domains conserved in all SEO proteins or in particular phylogenetic clades. We identified a 'potential thioredoxin fold' domain common to all SEO proteins, which is also found in enzymes that catalyze disulfide bond formation [28]. However, the canonical thioredoxin fold contains two central cysteine residues that are not present in the SEO proteins, indicating some functional divergence. Interestingly, thioredoxin folds lacking cysteines have also been found in other calcium-binding proteins such as calsequestrin [29]. Calsequestrin has three such domains that condense to form an acidic platform for high-capacity but low-affinity calcium adsorption that is most likely non-specific [30]. Calcium binding in forisomes is also weak and probably non-specific given that other divalent cations can also induce the typical conformational change [31,12]. The core of the calsequestrin thioredoxin fold domain is a five-strand β-sheet sandwiched by four α-helices [29]. A similar arrangement of α-helices and β-sheets is predicted within the thioredoxin domain of all SEO proteins analyzed with I-TASSER, which suggests that the modified thioredoxin fold could also be involved in calcium binding and the subsequent dispersion of forisomes and other P-proteins. Although thioredoxin folds are found in many different proteins, the presence of this domain together with the two PfamB domains we identified seems to be a unique characteristic of SEO proteins. Single PfamB domains were also identified in *Fabaceae *non-SEO proteins, but not in non-*Fabaceae *plants, which suggests the transfer of these domains from *SEO *to non-*SEO *genes by domain rearrangement [32,33].

All the SEO proteins contain the conserved C-terminal motif M1, which is characterized by four spatially conserved cysteine residues (Figure 6B). Although these residues could form disulfide bridges, this requires an oxidizing environment and disulfide bridges are generally not present in cytosolic proteins [34]. However, given the unique function of P-proteins and forisomes, it is possible disulfide bridges could form when the redox state of the cytosol is disrupted by cellular damage, stabilizing the dispersed state of SEO proteins and allowing them to seal off the injured sieve element. Indeed, forisome reactivity declines significantly in the presence of oxygen [30]. We identified several other motifs that were representative of SEO protein subgroups, e.g. motif M4 with potential intrinsic disorder, found at the N-terminus of subgroups 5 and 6 (Figure 6B). Disordered regions do not have a fixed three-dimensional structure, but can be involved in a variety of different molecular processes such as DNA/RNA or protein binding [35]. However, their precise role(s) in SEO proteins remains unclear.

**Figure 8:**
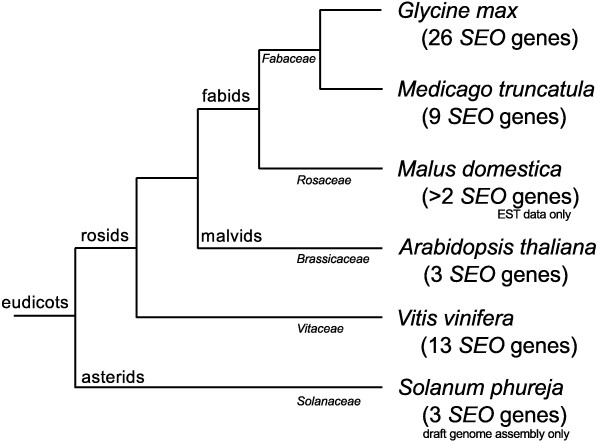
**Phylogenetic tree of the plants included in this investigation**. Phylogenetic tree of the plants included in this investigation according to the Angiosperm Phylogeny Group [75]. Clades and families are shown within the tree, and the numbers of known *SEO *genes are indicated.

To investigate the evolution of the *SEO *gene family, we studied the distribution and organization of *SEO *genes in *Fabaceae *and non-*Fabaceae *genomes (Figure 7 and Additional file 5) and their phylogenetic division into seven subgroups (Figures 1 and 8). Because subgroup 5 contains *SEO *genes from all the dicotyledonous plants with sequenced genomes included in our investigation, it is likely a similar ancestral *SEO *gene pre-dated the split between the rosids and asterids. Subgroup 4 contains only *SEO *genes from the closely-related plant families *Fabaceae *and *Rosaceae*, which suggests the subgroup 4 genes evolved by duplication and mutation prior to the divergence of the two plant families. With the exception of *MtSEO-F3*, all *SEO-F *genes cluster in the *Fabaceae*-specific subgroup 1 indicating they are unique within the plant kingdom. The function of the *GmSEO *genes clustering in subgroups 2 and 3 remains unclear. Although showing significant similarity to *SEO-F *genes, the corresponding proteins were not detected in forisomes. However, their detection might be affected by a low abundance in forisomes.

The distribution and organization of *SEO *genes in *M. truncatula *and *G. max *genomes indicates that several gene duplication events have occurred during the evolution of the *Fabaceae SEO-F *genes. With one known exception, *M. truncatula *SEO genes are clustered on chromosome 1 (the location of *MtSEO*-*F3 *is unknown), suggesting proliferation through tandem duplication. There is evidence that most *Fabaceae *share a common whole genome duplication event, which occurred approximately 50-60 million years ago, before *G. max *and *M. truncatula *diverged from a common ancestor [36,37]. Although this event cannot be verified by analyzing the position of *SEO *genes in the *M. truncatula *genome, it is possible that the evidence has been obscured by incidental gene loss events, since orthologs of several of the *SEO *genes present in *G. max *and other plants are missing in *M. truncatula*. For *G. max*, an additional whole genome duplication event is thought to have occurred ~15 million years ago [38,39]. This is supported by the observed synteny in the *SEO *gene clusters on *M. truncatula *chromosome 1, *G. max *chromosome 10, and the paralogs on *G. max *chromosome 20 (Figure 7). Definitive *G. max *orthologs could be identified for five of the nine *M. truncatula SEO *genes, and most of them exist as paralogs in *G. max*, leading to the conclusion that they were present prior to the split of the two *Fabaceae *and therefore the duplication event in *G. max*. No equivalent to the *SEO *gene cluster in *A. thaliana *or the arrangement of *SEO *genes in *V. vinifera *could be identified in *G. max *or *M. truncatula*, as their orthologs are not organized in clusters in both *Fabaceae*. Therefore it seems likely that additional and independent gene duplication and reorganization events affected the *SEO *genes in *A. thaliana *and *V. vinifera*. We also identified a number of potential *SEO *pseudogenes, five of which are found in *G. max*, which probably could be maintained because of the functional redundancy created by gene duplication. Nevertheless, we cannot exclude the possibility that at least the expressed pseudogenes ('pot. ψe') have evolved to take on novel functions, such as the regulation of gene expression [40,41]. *SEO *genes appear to be widespread in dicotyledonous plants and may therefore provide a powerful new tool to study the evolution of gene families in dicotyledons.

## Conclusions

We provide evidence that *SEO *genes are widely distributed in non-*Fabaceae *species and most probably encode P-proteins. The strong conservation of the gene structure, protein motifs and domains, the phylogenetic profile and the genomic synteny indicate a common phylogenetic origin for all *SEO *genes. Numerous tandem gene and whole genome duplication events appear to have contributed to the evolution of forisome genes in *Fabaceae*. We identified a fourth *M. truncatula *gene encoding a forisome component and presented the first analysis of forisome genes in *G. max*.

## Methods

### Identification of gene family members

Protein models were obtained from the sequenced genomes of *Medicago truncatula *[42], *Glycine max *[43], *Arabidopsis thaliana *[44], *Vitis vinifera *[45], *Oryza sativa *[46], *Sorghum bicolor *[47], *Zea mays *[48], *Brachypodium distachyon *[49] and *Physcomitrella patens *[50]. In addition, we used a *Malus domestica *EST collection from the "National Center for Biotechnology Information" and the not yet annotated genome sequence from *Solanum phureja *[51]. With the previously published forisome proteins MtSEO1, MtSEO2 and MtSEO3 [14,15] a BLASTP search was carried out against the protein annotations. Hits with significant similarities (E values lower than 1e-10) were analyzed for global similarity by aligning with the MtSEO proteins. Proteins with only local sequence similarities were not added to the SEO family. To identify further members the BLASTP search was repeated with the newly identified proteins. In addition and in case of missing protein annotations (EST data, non-annotated genomes), the full-length cDNA sequences of all identified *SEO *genes were used in a BLASTN search (threshold E < 0.001). Falsely-annotated *SEO *genes indicated e.g. by the presence of shortened open reading frames were re-annotated from genomic sequences with FGENESH or by aligning the genomic sequence with cDNA sequences of other *SEO *genes. For the identification of correctly spliced cDNA sequences, three independent cDNAs from total seedling RNA (see subheading "Expression analysis") were used for full-length amplification of the corresponding *SEO *genes using the oligonucleotides listed in Additional file 6. Sequence-verified *SEO *genes were deposited in GenBank (accession numbers are listed in Additional file 1). The exon-intron structure of the *SEO *genes was identified by comparing genomic and cDNA sequences. For *MtSEO-F3*, and the *MdSEO *and *SpSEO *genes, for which no or only preliminary genomic sequence data were available, genomic clones were amplified *de novo *by PCR.

### Forisome isolation and peptide sequencing

Forisomes were isolated from purified *M. truncatula *and *G. max *phloem tissue (see next section) by density gradient centrifugation according to established protocols [12]. After fractionation by SDS-PAGE the major 75-kDa protein band was excised from the gel matrix, purified and subsequently characterized by ESI-MS/MS as described [13,14]. The resulting peptide masses were screened against a database containing the SEO proteins and only those peptides unique for a single SEO protein were considered for further analysis.

### Expression analysis

Phloem-enriched tissue was prepared from *S. phureja *and *G. max *cv. Williams 82 by scraping the inner side of peeled stem rinds with a scalpel. The remaining stem rind was used as phloem-deficient material. *M. truncatula *cv. Jemalong A17 phloem was enriched by cutting stems in half longitudinally, removing the pith and scraping off the cortex with a scalpel. The cortex of the stem was used as phloem-deficient material. For *A. thaliana *cv. Col-0 phloem-enriched tissue was obtained by cutting out midribs from young leaves. For control experiments with phloem-deficient material we used parts of leaves lacking visible veins.

Total RNA was isolated from tissues ground to powder under liquid nitrogen using the NucleoSpin RNA^® ^Plant Kit (Macherey-Nagel, Düren, Germany). Total RNA was reverse transcribed with SuperScript II (Invitrogen, Karlsruhe, Germany) following the manufacturer's instructions. For all PCRs, partial but intron-spanning parts of the *SEO *genes were amplified using the oligonucleotides listed in Additional file 6. The integrity of all PCR products was verified by sequence analysis. If no products were generated, additional PCRs were performed using different combinations of primers on cDNA derived from young and old leaves, shoots, roots, buds and flowers. Only if no product was detected in this additional experiment were the corresponding *SEO *genes designated as potential pseudogenes. Expressed *SEO *genes containing frameshift mutations were designated as potential expressed pseudogenes. The expression of *M. truncatula GAPDH *[52], *A. thaliana ACT2 *[53], *S. phureja GAPDH *[54] and the *G. max *F-box gene [55] were used as positive controls.

### Promoter analysis

The P*AtSEOa*-GFP_ER _construct was generated by amplifying a 997-bp *AtSEOa *promoter-specific fragment (P*AtSEOa*) from *A. thaliana *genomic DNA, while the P*GmSEO-F1*-GFP_ER _construct was generated by amplifying a 2500-bp *GmSEO-F1 *promoter-specific fragment (P*GmSEO-F1*) from *G. max *genomic DNA (oligonucleotides listed in Additional file 6). Both PCR products were digested with *Kpn*I and *Xho*I and inserted into the corresponding restriction sites of pBSGFP_ER_, containing the downstream ER-targeted GFP coding region [14]. The promoter-GFP_ER _constructs were then excised and transferred into the *Kpn*I/*Hin*dIII sites of the binary vector pBIN19 [56] to obtain pBP*AtSEOa*-GFP_ER _and pBP*GmSEO-F1*-GFP_ER_, respectively. The binary vector pBP*AtSEOa*-GFP_ER _was introduced into *Agrobacterium tumefaciens *LBA4404 [57] and transformation of *A. thaliana *was carried out by floral dip [58]. Seeds were sterilized and germinated on Murashige and Skoog medium [59] supplemented with 50 μg/ml kanamycin for the selection of transgenic plants. GFP_ER _expression was monitored in transverse and longitudinal stem sections by confocal laser scanning microscopy (CLSM; Leica TCS SP5 X, Wetzlar, Germany; excitation 488 nm, emission 500-600 nm). Sieve plates were stained with a 0.01% aniline-blue solution according to established protocols [60] and visualized by CLSM (excitation 364 nm, emission 470-530 nm). The binary vector pBP*GmSEO-F1*-GFP_ER _was introduced into *A. rhizogenes *strain NCPPB2659 and transgenic *G. max *roots were obtained following established protocols [61]. Longitudinal sections of the roots were analyzed as above.

### Phylogenetic analysis

The OrthoMCL program [62] was used to cluster SEO proteins into subgroups, with an inflation parameter of 3. The protein sequences were aligned with T-Coffee [63] and the alignment was end trimmed to start and end with the domains predicted for all SEO proteins (SEO-NTD and SEO-CTD). The optimal evolutionary model for the family was calculated from this alignment with ProtTest [64]. RAxML [65] was used for tree building with the evolutionary model parameter JTT+F+I+G and a bootstrap of 1000. The best tree was visualized with FigTree [66].

### Domain analysis

Domain annotation was achieved by screening the NCBI Conserved Domain Database (v2.17) [21] in combination with CD-Search [67] and the PfamA and PfamB databases v23.0 [20], using a significance threshold of 1e-05. Domain arrangements were visualized using Jangstd [68]. HMMER 3.0 beta 2 was used to construct Hidden Markov Models (HMMs) and carry out searches [69]. Unique motifs in the SEO family were identified by extracting partial alignments to construct HMMs. Three dimensional protein structure prediction was carried out with I-TASSER [70]. The resulting protein structures were compared to structures in the Protein Data Bank (PDB) using the program TM-align [71]. Sequence logos of alignments were generated with WebLogo [72]. To identify regions of disorder, an in-house database was created by scanning protein sequences from annotated plant genomes with VSL2B [73]. Disordered sequences with a length of at least 20 amino acids were clustered with cd-hit [74], aligned and used to build HMMs. Disordered regions in the SEO family were predicted with this set of HMMs. All models were tested against SEO proteins as well as all available protein predictions for the plants included in our investigation.

## Authors' contributions

BR identified the *SEO *gene family members, cloned the *MtSEO *and *SpSEO *genes, performed the computational and domain analyses and drafted the manuscript. AME identified and cloned the *GmSEO *genes, isolated forisomes from *G. max *and *M. truncatula*, performed the P*GmSEO-F1 *promoter studies and participated in drafting the manuscript. SBJ cloned the *AtSEO *genes and performed the P*AtSEOa *promoter studies. SN participated in cloning the *GmSEO *genes. ARR supported the computational analyses and built the disordered region database. BM participated in analyzing the potential thioredoxin fold. EBB conceived the computational analyses and helped drafting the manuscript. DP participated in conceiving, design and coordination of this study and revised the manuscript. GAN conceived the study, participated in its design and helped to draft the manuscript. All authors read and approved the final manuscript.

## Supplementary Material

Additional file 1 **Table of all *SEO *genes included in this investigation**. Summary of all known *SEO *genes identified in *Medicago truncatula *(Mt), *Glycine max *(Gm), *Malus domestica *(Md), *Arabidopsis thaliana *(At), *Vitis vinifera *(Vv), *Solanum phureja *(Sp), *Vicia faba *(Vf), *Pisum sativum *(Ps) and *Canavalia gladiata *(Cg). The E values for the different protein products result from a BLASTp search with MtSEO-F1 against a protein database containing all identified SEO proteins. Click here for file

Additional file 2 **Table of peptide sequences obtained from forisomes**. Assigned peptide sequences generated by ESI-MS/MS from purified forisomes from *Medicago truncatula *(Mt) and *Glycine max *(Gm). Click here for file

Additional file 3 **Table with results for structure prediction of the potential thioredoxin fold with I-TASSER and TM-align**. Accuracy of the predicted thioredoxin fold structure of MtSEO and AtSEO proteins with I-TASSER, and of the TM-alignment with tryparedoxin II. Click here for file

Additional file 4 **Table of non-SEO proteins containing SEO domains**. List of predicted non-SEO proteins from *Medicago truncatula *(Medtr) and *Glycine max *(Glyma) carrying the SEO N-terminal domain (NTD) or SEO C-terminal domain (CTD). Click here for file

Additional file 5 **Chromosomal organization of the *SEO *genes**. Schematic overview of *SEO *chromosomal loci in the plants included in this investigation. Orthologs between *MtSEO *and *GmSEO *genes as well as *SEO *paralogs in *G. max *are marked with matching symbols. Chromosomes and genes are not drawn to scale. Click here for file

Additional file 6 **List of oligonucleotides**. Click here for file
