# Supplementary material for: Molecular and phylogenetic characterization of the sieve element occlusion gene family in Fabaceae and non-Fabaceae plants
Source: BMC Plant Biol. 2010 Oct 8;10:219. doi: 10.1186/1471-2229-10-219 (PMC3017817; doi:10.1186/1471-2229-10-219)
Supplement: Additional file 1 — Table of all SEO genes included in this investigation. Summary of all known SEO genes identified in Medicago truncatula (Mt), Glycine max (Gm), Malus domestica (Md), Arabidopsis thaliana (At), Vitis vinifera (Vv), Solanum phureja (Sp), Vicia faba (Vf), Pisum sativum (Ps) and Canavalia gladiata (Cg). The E values for the different protein products result from a BLASTp search with MtSEO-F1 against a protein database containing all identified SEO proteins. [file 1471-2229-10-219-S1.PDF]

gene name	transcript identified	GenBank Accession	length ORF	gene locus in genome annotation	E value blastp with MtSEO-F1
MtSEO-F1	yes	EU016204	1941 bp	Medtr1g089750	0
MtSEO-F2	yes	HM162875	2025 bp	Medtr1g089740	0
MtSEO-F3	yes	HM162876	2103 bp	*not in genome assembly*	1E-167
MtSEO-F4	yes	HM162877	2013 bp	Medtr1g089720	0
MtSEOa	yes	HM162878	1992 bp	Medtr1g089610 + Medtr1g089600	2E-086
MtSEOb	yes	HM162879	2007 bp	Medtr1g089570	6E-090
MtSEOc	yes	HM162880	2013 bp	Medtr1g089550	2E-091
MtSEOd	*no, pot. ψ*			Medtr1g089790	6E-075
MtSEOe	yes	HM162881	2187 bp	Medtr7g032330	3E-049
GmSEO-F1	yes	HM162862	1968 bp	Glyma10g32930	0
GmSEO-F2	yes	HM162863	1986 bp	Glyma10g32940	1E-174
GmSEO-F3	yes	HM162860	2001 bp	Glyma10g00250	0
GmSEO-F4	yes	HM162864	1998 bp	Glyma10g32950	0
GmSEOa	yes	HM162856	2094 bp	Glyma02g00280	1E-167
GmSEOb	*no, pot. ψ*			*not annotated, between 02g00280 and 02g00290*	3E-041
GmSEOc	yes	HM162857	2094 bp	Glyma04g36440	7E-043
GmSEOd	yes	HM162858	2115 bp	Glyma06g18430	1E-042
GmSEOe	yes	HM162859	2109 bp	Glyma08g13870	3E-046
GmSEOf	yes	HM162861	2094 bp	Glyma10g00260	1E-163
GmSEOg	yes	HM162865	2007 bp	Glyma10g32970	8E-091
GmSEOh	*yes; no frame, pot. ψe*			Glyma10g32980	6E-076
GmSEOi	yes	HM162866	2031 bp	Glyma11g37700	1E-024
GmSEOj	yes	HM162867	2073 bp	Glyma13g03610	1E-145
GmSEOk	*no, pot. ψ*			Glyma13g03620	9E-097
GmSEOl	yes	HM162868	2058 bp	Glyma13g26030	1E-137
GmSEOm	yes	HM162869	2181 bp	Glyma16g07300	2E-050
GmSEOn	*no, pot. ψ*			Glyma18g01620	4E-012
GmSEOo	yes	HM162870	2058 bp	Glyma20g11990	1E-138
GmSEOp	yes	HM162871	2079 bp	Glyma20g12020	1E-143
GmSEOq	*no, pot. ψ*			Glyma20g34650	1E-024
GmSEOr	yes	HM162872	2007 bp	Glyma20g34660	1E-091
GmSEOs	yes	HM162873	2007 bp	Glyma20g34670	1E-096
GmSEOt	*no, pot. ψ*			Glyma20g34700	8E-085
GmSEOu	yes	HM162874	1986 bp	Glyma20g34710	1E-175
GmSEOv	*yes; no frame, pot. ψe*			Glyma20g34720	0
MdSEOa	yes	HM162887	2043 bp	*EST data only*	8E-090
MdSEOb	yes	HM162888	2046 bp	*EST data only*	2E-097
AtSEOa	yes	HM162885	2466 bp	At3g01670	3E-036
AtSEOb	yes	HM162886	2220 bp	At3g01680	5E-049
AtSEOc	*no, pot. ψ*			At1g67790	8E-011
VvSEOa	*not determined*			GSVIVP00015934001	4E-040
VvSEOb	*not determined*			GSVIVP00020609001	2E-055
VvSEOc	*not determined*			GSVIVP00020610001	6E-055
VvSEOd	*not determined*			GSVIVP00020612001+GSVIVP00020613001	8E-061
VvSEOe	*not determined*			GSVIVP00020614001	9E-061
VvSEOf	*not determined*			GSVIVP00020615001	1E-056
VvSEOg	*not determined*			GSVIVP00020618001+GSVIVP00020619001	5E-059
VvSEOh	*not determined*			GSVIVP00020621001	5E-061
VvSEOi	*not determined*			GSVIVP00020622001	6E-045
VvSEOj	*not determined*			GSVIVP00029288001	4E-030
VvSEOk	*not determined*			GSVIVP00030721001	2E-044
VvSEOl	*not determined*			GSVIVP00030722001	6E-059
VvSEOm	*not determined*			GSVIVP00030723001	6E-025
SpSEOa	yes	HM162882	2169 bp	*genome not annotated*	1E-040
SpSEOb	yes	HM162883	2469 bp	*genome not annotated*	3E-043
SpSEOc	yes	HM162884	2457 bp	*genome not annotated*	3E-045
VfSEO-F1	yes	ABV32454	2052 bp	*no genome sequence available*	0
PsSEOa	yes	ACV83947	2055 bp	*no genome sequence available*	0
CgSEOa	yes	ABV32453	2004 bp	*no genome sequence available*	0
