# Supplementary material for: Molecular and phylogenetic characterization of the sieve element occlusion gene family in Fabaceae and non-Fabaceae plants
Source: BMC Plant Biol. 2010 Oct 8;10:219. doi: 10.1186/1471-2229-10-219 (PMC3017817; doi:10.1186/1471-2229-10-219)
Supplement: Additional file 2 — Table of peptide sequences obtained from forisomes. Assigned peptide sequences generated by ESI-MS/MS from purified forisomes from Medicago truncatula (Mt) and Glycine max (Gm). [file 1471-2229-10-219-S2.PDF]

protein	assigned peptide sequences
MtSEO-F1	YIFIYGGSDK
MtSEO-F1	WQIDSVEEYLK
MtSEO-F1	NGDNLLIYDGTTK
MtSEO-F2	VYLTHVNDNMK
MtSEO-F2	YIFIYGGNDPK
MtSEO-F2	NNPTEIPYFWMGIDGR
MtSEO-F3	STAVADSVASK
MtSEO-F3	TGTPVGLVEDR
MtSEO-F3	QAIADYNSLVK
MtSEO-F4	WYVVEYFSELPGLK
MtSEO-F4	WIQDFTLELEK
GmSEO-F1	ILWIPIVDTWDDKQK
GmSEO-F1	FNYLGKPIAPVLTPLGDR
GmSEO-F1	YTLAEFDTWKDK
GmSEO-F2	QDPSGWVVLSR
GmSEO-F2	WFWNLLEQTDDNAK
GmSEO-F2	ILGHAEPMYQTVLDFEK
GmSEO-F3	QDTQGWAILSK
GmSEO-F3	VYVSHTYDDEMFDNEPLFNVVSNIIK
GmSEO-F3	ILGHGQPMYQTVADFEK
GmSEO-F4	IFEGTNLVTK
GmSEO-F4	GELNNSVPSFWIGVER
GmSEO-F4	ADIIIENYQLGK
