# Supplementary material for: Molecular and phylogenetic characterization of the sieve element occlusion gene family in Fabaceae and non-Fabaceae plants
Source: BMC Plant Biol. 2010 Oct 8;10:219. doi: 10.1186/1471-2229-10-219 (PMC3017817; doi:10.1186/1471-2229-10-219)
Supplement: Additional file 3 — Table with results for structure prediction of the potential thioredoxin fold with I-TASSER and TM-align. Accuracy of the predicted thioredoxin fold structure of MtSEO and AtSEO proteins with I-TASSER, and of the TM-alignment with tryparedoxin II. [file 1471-2229-10-219-S3.PDF]

protein	Accuracy of model 1			Accuracy of TM-alignment of model 1 with Tryparedoxin II	
	C-Score	TM-Score	RMSD (Å)	TM-Score	RMSD
MtSEO-F1	-0.11	0.70 +/- 0.12	4.7 +/- 3.1	0.8365	1.38
MtSEO-F2	-0.42	0.66 +/- 0.13	5.4 +/- 3.4	0.7994	1.48
MtSEO-F3	-0.11	0.70 +/- 0.12	4.7 +/- 3.1	0.8297	1.38
MtSEO-F4	-0.01	0.71 +/- 0.11	4.5 +/- 3.0	0.8128	1.24
MtSEOa	0.45	0.77 +/- 0.10	3.5 +/- 2.4	0.8978	1.00
MtSEOb	0.40	0.77 +/- 0.10	3.6 +/- 2.5	0.8971	1.02
MtSEOc	0.31	0.75 +/- 0.10	3.7 +/- 2.5	0.8998	1.06
MtSEOe	0.11	0.73 +/- 0.11	4.3 +/- 2.8	0.8452	1.07
AtSEOa	0.20	0.74 +/- 0.11	4.0 +/- 2.7	0.8778	1.13
AtSEOb	-0.10	0.70 +/- 0.12	4.8 +/- 3.1	0.7963	1.31
