# Supplementary material for: Molecular and phylogenetic characterization of the sieve element occlusion gene family in Fabaceae and non-Fabaceae plants
Source: BMC Plant Biol. 2010 Oct 8;10:219. doi: 10.1186/1471-2229-10-219 (PMC3017817; doi:10.1186/1471-2229-10-219)
Supplement: Additional file 4 — Table of non-SEO proteins containing SEO domains. List of predicted non-SEO proteins from Medicago truncatula (Medtr) and Glycine max (Glyma) carrying the SEO N-terminal domain (NTD) or SEO C-terminal domain (CTD). [file 1471-2229-10-219-S4.PDF]

gene name in genome annotation	PfamB domain	SEO domain
Medtr3g067870	PB013523	NTD
Medtr3g102630	PB006891	CTD
Medtr4g018450	PB013523	NTD
Medtr8g073800	PB013523	NTD
Glyma06g37760	PB013523	NTD
Glyma06g37860	PB013523	NTD
Glyma10g10970	PB013523	NTD
Glyma14g29080	PB013523	NTD
Glyma20g16000	PB006891	CTD
