# Supplementary material for: Molecular and phylogenetic characterization of the sieve element occlusion gene family in Fabaceae and non-Fabaceae plants
Source: BMC Plant Biol. 2010 Oct 8;10:219. doi: 10.1186/1471-2229-10-219 (PMC3017817; doi:10.1186/1471-2229-10-219)
Supplement: Additional file 5 — Chromosomal organization of the SEO genes. Schematic overview of SEO chromosomal loci in the plants included in this investigation. Orthologs between MtSEO and GmSEO genes as well as SEO paralogs in G. max are marked with matching symbols. Chromosomes and genes are not drawn to scale. [file 1471-2229-10-219-S5.PDF]

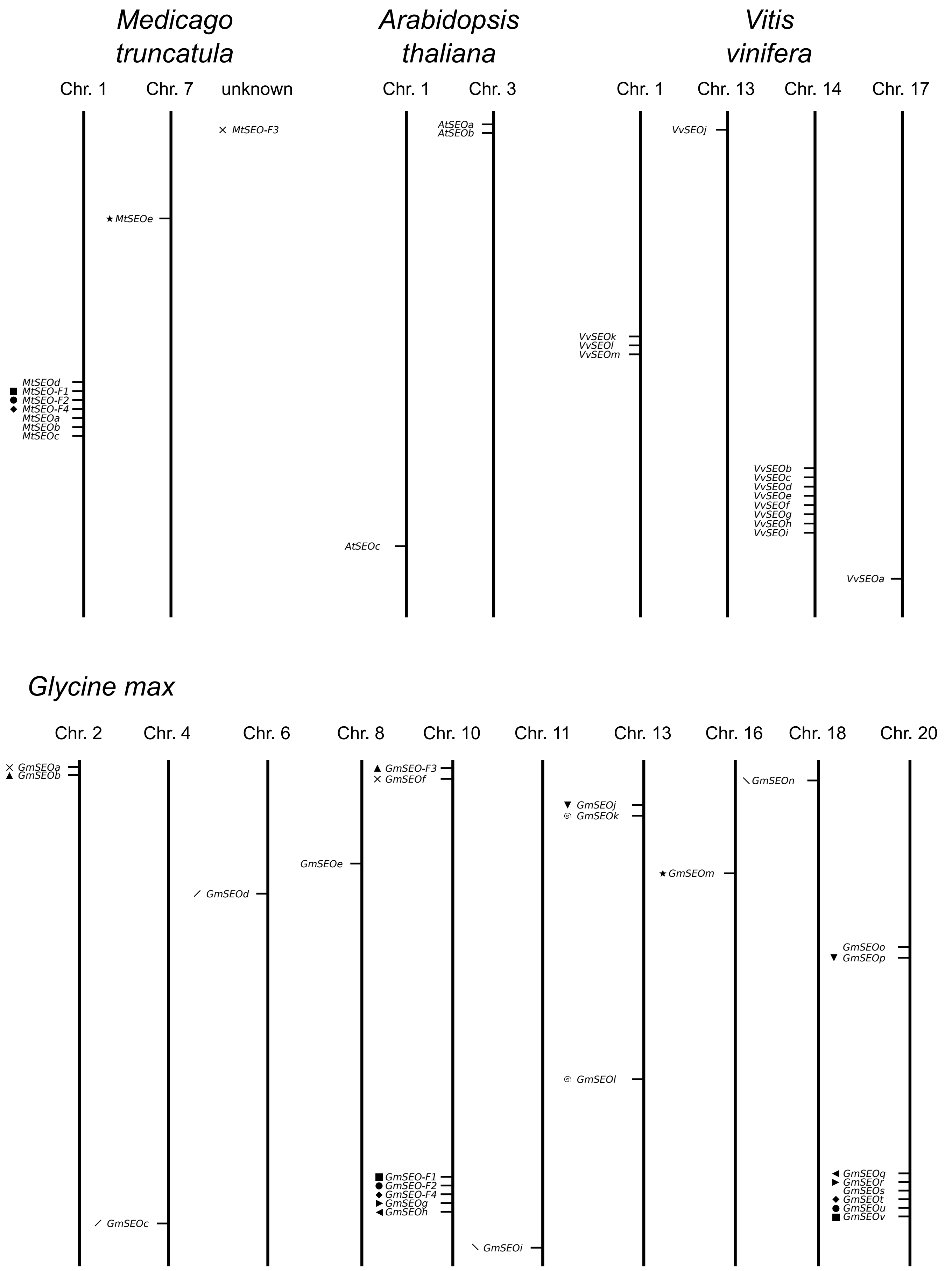
Medicago truncatula
Chr. 1
Chr. 7
unknown
MtSEO-F3
MtSEOe
MtSEOd
MtSEO-F1
MtSEO-F2
MtSEO-F4
MtSEOa
MtSEOb
MtSEOc
Arabidopsis thaliana
Chr. 1
Chr. 3
AtSEOa
AtSEOb
AtSEOc
Vitis vinifera
Chr. 1
Chr. 13
Chr. 14
Chr. 17
VvSEOj
VvSEOk
VvSEOl
VvSEOm
VvSEOb
VvSEOc
VvSEOd
VvSEOe
VvSEOf
VvSEOg
VvSEOh
VvSEOi
VvSEOa
Glycine max
Chr. 2
Chr. 4
Chr. 6
Chr. 8
Chr. 10
Chr. 11
Chr. 13
Chr. 16
Chr. 18
Chr. 20
GmSEOa
GmSEOb
GmSEO-F3
GmSEOf
GmSEOn
GmSEOj
GmSEOk
GmSEOe
GmSEOm
GmSEOd
GmSEOo
GmSEOp
GmSEOl
GmSEO-F1
GmSEO-F2
GmSEO-F4
GmSEOg
GmSEOh
GmSEOq
GmSEOr
GmSEOs
GmSEOt
GmSEOu
GmSEOv
GmSEOc
GmSEOi
