# Supplementary material for: Molecular and phylogenetic characterization of the sieve element occlusion gene family in Fabaceae and non-Fabaceae plants
Source: BMC Plant Biol. 2010 Oct 8;10:219. doi: 10.1186/1471-2229-10-219 (PMC3017817; doi:10.1186/1471-2229-10-219)
Supplement: Additional file 6 — List of oligonucleotides. [file 1471-2229-10-219-S6.PDF]

Name	Oligonucleotide sequence 5'-3' (restriction sites underlined)
PAtSEOa fw KpnI	AGAGGTACCTCAGCCGGAGATCATCC
PAtSEOa bw XhoI	AGACTCGAGATTGGCGAGGTTGAGAG
PGmSEO-F1 fw KpnI	AGAGGTACCTCATAGAAAGTAAGAAATTAAAG
PGmSEO-F1 bw XhoI	AGACTCGAGGATGATGGTGGTGATAATTC
MtSEO-F1 fw	ATGTCATTGTCCAATGGAAC
MtSEO-F1 bw	TCATATCTTGCCATTCTGTG
MtSEO-F1 fw internal	CCAGTGTTGAGCCCTTTAG
MtSEO-F1 bw internal	GAGCAGCAGCATCGTCAC
MtSEO-F2 fw	ATGTCCACTGCATTGTCC
MtSEO-F2 bw	TCAAATGCAGCAACTATC
MtSEO-F2 fw internal	CAGGGCGTGGGATAATAAAGA
MtSEO-F2 bw internal	CCATCACACGTCCACAAAAAG
MtSEO-F3 fw	ATGTCGTCTTCAATGGC
MtSEO-F3 bw	TCAAGACCTTTTCTCAATC
MtSEO-F3 fw internal	GGACCCCCTTGTGTTTG
MtSEO-F3 bw internal	TAAGCTTTCTATGCCTATCCA
MtSEO-F4 fw	ATGTCCCTTTCCAACTTAG
MtSEO-F4 bw	TCAAACACCAAGATTGTTTG
MtSEO-F4 fw internal	CTAGCCGCACGAATAACT
MtSEO-F4 bw internal	ATCATCTTTGCCCACTTG
MtSEOa fw	ATGATTCGCCTATCAAGATC
MtSEOa bw	CTAACCATTATGGCAGC
MtSEOa fw internal	ATGATAGGGGGAGGAAGA
MtSEOa bw internal	TGAGAGCAAATATGAACAGAA
MtSEOb fw	ATGGTTAGCCTAGTGCG
MtSEOb bw	CTAATGTTCAGCATTAACC
MtSEOb fw internal	CACCAATTAAAGGCATCAA
MtSEOb bw internal	AAATCTGGAATTTTCCCAG
MtSEOc fw	ATGGCCACCATCATCAAAG
MtSEOc bw	CTAATGTTTTCCATTTGCAT
MtSEOc fw internal	TTGCTTTATGATGGTGCTAC
MtSEOc bw internal	CATCGGTTGTTTTGTGAAT
MtSEOd fw internal	ACGCTCCAAAAGAATCC
MtSEOd bw internal	GCCAACAGCAATCACAA
MtSEOd fw internal 2	AAACCCCGACTGAAGTC
MtSEOd bw internal 2	AAACTTGGTGAATTCTTGG
MtSEOe fw	ATGACTAGCATTGGCAAG
MtSEOe bw	TTAATTAGTATTAGGAGGAG
MtSEOe fw internal	GTCTCAAATCATATCCCAGTT
MtSEOe bw internal	TTCCCCAAATCCACATC
MtGAPDH fw	TTTGGTTGCTAGAGTTGC
MtGAPDH bw	GGGAGCATCCTTACTAG
GmSEO-F1 fw	ATGTCACTGTCCAATGG
GmSEO-F1 bw	TCAGATGTTGCCATTGTTTG
GmSEO-F1 fw internal	ATAGATTGCAGGAGAATCC
GmSEO-F1 bw internal	ACAAGCACATCTGCATTG
GmSEO-F2 fw	ATGGCGTGGGTACTGTC
GmSEO-F2 bw	TCAGAGATTGCAGCTGTTTG
GmSEO-F2 fw internal	AATAACGGGTTGCAAGAG
GmSEO-F2 bw internal	TGAAGTATGATCATATGGG
GmSEO-F3 fw	ATGTCGATCTCCAACAC
GmSEO-F3 bw	TTAGGTTGCGGTTGTGGAC
GmSEO-F3 fw internal	GATCCGAATGACAAGAGC
GmSEO-F3 bw internal	GCTAGGACATCCAAAGTG
GmSEO-F4 fw	ATGGCACAACTGTCCAATG
GmSEO-F4 bw	TCAACAGTTCAAAGCATCATC
GmSEO-F4 fw internal	TATATGAAAGATTGCAAGAG

GmSEO-F4 bw internal	CAATGACACTTGAAGCTG
GmSEOa fw	ATGGCATTAGTGACTTCAC
GmSEOa bw	TTAAGAAGCGTAGAGCTTC
GmSEOa fw internal	CGAGGGATTGCAGGAAG
GmSEOa bw internal	TGGGTGAAGAATATCAGC
GmSEOb fw internal	AAATTTCTGGAATCTTCAGC
GmSEOb bw internal	AATTCCTCTTTCTTGAAACC
GmSEOb fw internal 2	AACTCATTGAGACTGCTG
GmSEOb bw internal 2	AGCCGAATCTCGTCTTC
GmSEOc fw	ATGAGCAACATGAGCAAC
GmSEOc bw	TTACTCCACACAACAGCG
GmSEOc fw internal	TTGATAATTTGTACAAAGATG
GmSEOc bw internal	TGTTGAGATAATCATTCATTG
GmSEOd fw	ATGAGTAACCACACTCG
GmSEOd bw	TTACTCCACACAACAACG
GmSEOd fw internal	GGTACTTGACAATTTGTAC
GmSEOd bw internal	CACCGTCTTCCTCAATTC
GmSEOe fw	ATGGCAATGGTGCCCC
GmSEOe bw	TTACTCATTGCAGCAGCG
GmSEOe fw internal	TCAAGATTCAACCAGATTAG
GmSEOe bw internal	TGCTGCGTCTTTCCATTG
GmSEOf fw	ATGGCATTAGTGTCTTCAC
GmSEOf bw	TTAAGAAGCGTAGAGCTTC
GmSEOf fw internal	ATCTATGAGGGATTACAGG
GmSEOf bw internal	TATCAGCAGGGTAGTTAG
GmSEOg fw	ATGGCTGCCAAGCATTCACAAG
GmSEOg bw	CTAGTTTATGGCAATTGAAG
GmSEOg fw internal	TTATGATTCAATTAAAACTGG
GmSEOg bw internal	GTGTATGCAGCGATGATG
GmSEOh fw	ATGAGCATAATAATGTCGTC
GmSEOh bw	CTAGTAGTGAATGGTATTG
GmSEOh fw internal	TTCCAAACAGTTTATGATTC
GmSEOh bw internal	AACGGTGAGTGATGTCC
GmSEOi fw	ATGCAACATGATATTCTTATAAAG
GmSEOi bw	TCATGGCTGGTACACCAC
GmSEOi fw internal	ACTGGTCCAGCAAACATG
GmSEOi bw internal	TTCCTTGACTTGGTCTTTC
GmSEOj fw	ATGTCCAAGTCACTTTCAAG
GmSEOj bw	CTATGGTTCAAGACCATGG
GmSEOj fw internal	ATACGAGAAATTGAAGGAG
GmSEOj bw internal	ATCCGTGGGGTAGTTAG
GmSEOk fw internal	ATCGATCCATGTGAAATTG
GmSEOk bw internal	GGGTAGTTAGCAAATTCC
GmSEOk fw internal 2	TTTGCTCTGGAATATGGC
GmSEOk bw internal 2	CATTGCTGAGTGAGTCTG
GmSEOl fw	ATGTCTAAGCAAGTTTCCAG
GmSEOl bw	TTAGGCAATGTCACTGGCT
GmSEOl fw internal	AATGCGAAATTGAAGGAAG
GmSEOl bw internal	ATGTCAGTGGGGTAATTG
GmSEOm fw	ATGAATAGCATTGGCAAGC
GmSEOm bw	TCAAATGGTAGGAGGAGTAAC
GmSEOm fw internal	CGACGAGCTTCTGATTC
GmSEOm bw internal	TCAAACCTGCAGCAAGG
GmSEOn fw internal	TATATCGTGGTAATACTTTG
GmSEOn bw internal	GGTTTCTGAACAGCATTC
GmSEOn fw internal 2	TCTCTAGAGGAAACAAAGC
GmSEOn bw internal 2	ATTTTTTGTCAGATTCTTTGG
GmSEOo fw	ATGTCCCATTCACTTTCAAG
GmSEOo bw	CTATTTCTTACGGTCATGAC
GmSEOo fw internal	TACACGAGAAGTTGAAGG
GmSEOo bw internal	TCGTTCCAATATATCCATG
GmSEOp fw	ATGTCTAAGTCACTTTCAAAC

GmSEOp bw	CTATGGTTCAAGACCATGAC
GmSEOp fw internal	ATACGAGAAATTGAAGGAG
GmSEOp bw internal	TATCCGTTGGGTAGTTAG
GmSEOq fw internal	TGAACAGTATAAGATCGTG
GmSEOq bw internal	CCACTGTCTCCGAGATG
GmSEOq fw internal 2	TCCTCCCTAGACATCAC
GmSEOq bw internal 2	ATTTCAAGGTATGAGCAAC
GmSEOr fw	ATGGCTACCAAGCATTCAC
GmSEOr bw	CTAGTTTGAAGCAATTGAAG
GmSEOr fw internal	GAGGAAGAGATTTCAGTAC
GmSEOr bw internal	CTTCCCAGCAGCATTAG
GmSEOs fw	ATGAGCATTATCATGTCCAC
GmSEOs bw	CTAGTGAATGGCATTGGCA
GmSEOs fw internal	GGAAGAGATTTCAGTACTC
GmSEOs bw internal	GTAACTTCCCTGCAACG
GmSEOt fw internal	TTGCAAGAAGATCCAAAAG
GmSEOt bw internal	TCTATCGAACACTCTCTC
GmSEOt fw internal 2	GGATCTCCATTGTGGTTG
GmSEOt bw internal 2	TTTTGAAACTCAGCCACG
GmSEOu fw	ATGGCATTGGTACTGTCC
GmSEOu bw	TCAGAGATTGCAGCTGTTTG
GmSEOu fw internal	AAATAAAAGGTTTCAAAAAAGG
GmSEOu bw internal	AAGCACGGTTTGATACATG
GmSEOv fw	ATGTCACTGTCCAATGGAGA
GmSEOv bw	TCAGATGTTGACATTGTTCGT
GmSEOv fw internal	CATGACAGATTGCATGAG
GmSEOv bw internal	TAGCACATCTGCATTATTC
GmF-Box fw	GAAAACTGAATCGAACCAAA
GmF-Box bw	GGAGAGTACTTATTTGCTTA
MdSEOa fw	ATGCTAGGCATAGCAC
MdSEOa bw	TCAATGATGCGCGTTGATGG
MdSEOb fw	ATGCTAGGTCTAGCAAAC
MdSEOb bw	TTAGTGATGTGCAGTAGG
AtSEOa fw	ATGGCCCAACGCTTTCAATTG
AtSEOa bw	TTACTCAAGGCAGCATTGG
AtSEOa fw internal	AGTTCGAGGCCCTTCACATGAAC
AtSEOa bw internal	CGATGCTTTACTCACCAGTCC
AtSEOb fw	ATGGAGTCGCTGATCAAG
AtSEOb bw	TTAGAAGTTGTAGTTCTCGTC
AtSEOb fw internal	ATGGCGGCCAAAGCAACGGCCAA
AtSEOc fw internal	TTGAGCAGCTACTTCGTAGCC
AtSEOc bw internal	GCTGCATGCATGTGAGTGCAC
AtSEOc fw internal 2	CATGGATGGTCTATCAATCTC
AtSEOc bw internal 2	GCCGTGTGACTCAGTTCACAC
AtActin fw	CCTCATCATACTCGGCCTTGGAG
AtActin bw	GTAAGAGACATCAAGGAGAAGCTCTC
SpSEOa fw	ATGGCAAGTCATGCTTTG
SpSEOa bw	TCAATCAGTGCAGCAAC
SpSEOa fw internal	GTGGCCATCTTAAAACAAC
SpSEOa bw internal	TCCAATCCATGTCTTCTC
SpSEOb fw	ATGGCAAATGTTAACCCA
SpSEOb bw	TCACTCAATGCAGCAGC
SpSEOb fw internal	CAATATGGTGAATTTGGAT
SpSEOb bw internal	CACTAATCCAAGTGAAAAT
SpSEOc fw	ATGGCAAGTGTTAACCCA
SpSEOc bw	TCAATCAGTGCAGCAGC
SpSEOc fw internal	CAATATGGTGAATTTGGTC
SpSEOc bw internal	CACTAATCCAAGTGAAAAC
SpGAPDH fw	CAAGGACTGGAGAGGTGG
SpGAPDH bw	TTCACTCGTTGTCGTACC
